# Detecting and monitoring concerns against HPV vaccination on social media using large language models

**DOI:** 10.1038/s41598-024-64703-3

**Published:** 2024-06-21

**Authors:** Sunny Rai, Melanie Kornides, Jennifer Morgan, Aman Kumar, Joseph Cappella, Sharath Chandra Guntuku

**Affiliations:** 1 Department of Computer and Information Science, University of Pennsylvania, Philadelphia, PA 19104 USA; 2 School of Nursing, University of Pennsylvania, Philadelphia, PA 19104 USA; 3 Annenberg School of Communication, University of Pennsylvania, Philadelphia, PA 19104 USA; 4 Leonard Davis Institute of Health Economics, University of Pennsylvania, Philadelphia, PA 19104 USA

**Keywords:** Public health, Infectious diseases

## Abstract

Health risks due to preventable infections such as human papillomavirus (HPV) are exacerbated by persistent vaccine hesitancy. Due to limited sample sizes and the time needed to roll out, traditional methodologies like surveys and interviews offer restricted insights into quickly evolving vaccine concerns. Social media platforms can serve as fertile ground for monitoring vaccine-related conversations and detecting *emerging* concerns in a scalable and dynamic manner. Using state-of-the-art large language models, we propose a minimally supervised end-to-end approach to identify concerns against HPV vaccination from social media posts. We detect and characterize the concerns against HPV vaccination pre- and post-2020 to understand the evolution of HPV vaccine discourse. Upon analyzing 653 k HPV-related post-2020 tweets, *adverse effects, personal anecdotes,* and *vaccine mandates* emerged as the dominant themes. Compared to pre-2020, there is a shift towards personal anecdotes of vaccine injury with a growing call for parental consent and transparency. The proposed approach provides an end-to-end system, i.e. given a collection of tweets, a list of prevalent concerns is returned, providing critical insights for crafting targeted interventions, debunking messages, and informing public health campaigns.

## Introduction

One of the key national aims outlined in the Healthy People 2030 Framework, is to “Increase the proportion of adolescents who get recommended doses of the HPV vaccine—IID‑08”^1^. Motivation to vaccinate, which encompasses intersecting constructs of intention, willingness, acceptability, hesitancy, and the social environment (e.g. social norms about vaccination, provider recommendations, vaccine myths, and misinformation about vaccines), collectively contribute to vaccine uptake^2^. Notably, parental reluctance to vaccinate their children, known as parental vaccine hesitancy, significantly correlates with lower HPV vaccination rates among adolescents^3^. Research conducted on a national US sample revealed a significant prevalence (23%) of parental hesitancy toward HPV vaccination, which exerts a stronger influence on receiving the vaccine than barriers like cost or accessibility^4^. The reasons for vaccine hesitancy evolve with exposure to vaccine-related discourse^5^. Designing effective public health messaging and policies to mitigate vaccine hesitancy necessitates ongoing monitoring of emerging concerns impeding vaccine confidence^6^.

Prior works have primarily relied on interviews and surveys to uncover concerns against vaccines^7^; however, there is a growing interest in examining social media data due to its extensive reach among the masses^8^. Nearly all parents of adolescents engage with social media, with 68% using it for health information^9^. Manual annotation of social media posts permits examination of a limited number of posts and remains static. However, the concerns evolve, and dynamically tracking them is an unsolved research challenge. Social media is an easily accessible platform to learn people’s concerns and beliefs regarding vaccinations^10^. Vaccine misinformation (also concerns against vaccination) is a frequently studied research problem; forty-three percent of health-related misinformation studies on social media are vaccine-related^11^. Moreover, social media content discussing vaccine harms strongly influences vaccination behavior^12^. Individuals exposed to vaccination harm-related stories were 44% more likely to refuse vaccination and 13% more likely to delay vaccination than unexposed individuals^13^.

Digital public health surveillance via social listening is a promising avenue for identifying and addressing health concerns at the earliest stages^14^. WHO built a social listening platform, EARS to track emerging concerns related to COVID-19 from social media posts using a semi-supervised machine learning technique^15^. Sentiment^16^ and stances^17^ against HPV vaccination on social media platforms, including Twitter and Reddit^18^ are monitored to estimate people’s opinions. Topical analyses and the diffusion of HPV-related content on social media can reveal underlying concerns and prevent the cascading effect of misinformation^19,20^. However, social listening platforms need to be repetitively manually supervised to be effective^21^. This paper aims to overcome the need for periodic human supervision and proposes a minimally supervised approach to identify emerging concerns against HPV vaccination from public tweets. Our approach relies on semantic information encoded in language model embeddings and thus requires minimal labeled data. The proposed method is an end-to-end system wherein, given a set of tweets, it generates a set of articulated concerns directly applicable to subsequent tasks, such as crafting debunking messages or designing public health initiatives. Moreover, the existing public concerns collected from HPV-related online communication can be easily compared and contrasted with concerns identified from the latest ongoing dialogue on HPV vaccination, This will facilitate dynamic adjustments to debunking messages or enhancements to ongoing health campaigns based on the evolving landscape of concerns.

## Methods

### Data

Using Twitter API, we collected 653 K HPV-related tweets posted from Jan 2020 to June 2022 using the search keywords—{*hpv, gardasil, papilloma*}. The tweets were processed to mask the URLs and user mentions from the content of the tweets. The proposed approach comprises three phases (a) Characterizing HPV discourse, (b) Identifying topics associated with concerns against HPV vaccines and (c) Leveraging GPT-4 for contextual topic labeling. These phases are discussed in detail below.

### Characterizing HPV discourse

The first step identifies latent themes in HPV discourse using topic modeling. Latent Dirichlet allocation (LDA)^22^ is an unsupervised clustering algorithm to identify latent topics in large quantities of text. The algorithm assumes that each word occurrence can be attributed to one or more topics generated from the corpus. Words are assigned to a topic based on co-occurrence with other words across the corpus of HPV-related tweets and repeated until all words are designated to a set of topics with other semantically similar words. These topics represent semantically coherent clusters of words in which words are assigned weights based on their likelihood of occurring within each topic. We first removed the top 100 most frequent words in the dataset. Using the DLATK library’s interface for the MALLET implementation of LDA^23^, we generated 50, 100, and 150 topics, with an alpha level of 5. We computed coherence scores^24^ for all sets of topics (See Supplementary). Two human experts independently analyzed the quality of the word clusters. The number of topics was set to 100 after evaluating the quality and granularity of topics.

### Topics associated with “concern” specific HPV discourse

To recognize topics related to concerns against HPV vaccines, one alternative is to manually analyze the topics (cluster of words) and related tweets. However, it is a slow and labor-intensive process that needs to be performed after every few months^21^. Automation will enable periodic and fine-grained analysis of vaccine hesitancy at scale. In our approach, we first build a classifier to automatically label tweets expressing concern against HPV vaccination and then perform a regression analysis on topic distribution (in Sect. "Characterizing HPV discourse") of labeled tweets to identify topics correlated with concerns.

We used an existing hand-labeled collection of 3876 tweets^8^ posted between December 15, 2019, and March 31, 2020, for training a supervised classifier for HPV vaccination related concern detection. 24% of 3876 tweets were hand-coded as expressing concern against HPV vaccines or vaccination. Here, *concern* refers to any reason (misinformation-driven or legitimate) causing reluctance against HPV vaccines. The prediction performance of a classifier hinges on access to abundant labeled data within the specific domain. However, annotating large amounts of data is both time-consuming and cost-intensive. We mitigate these challenges by using contextual embeddings from language models. Compared to word embedding such as word2vec, contextual embeddings are adept at disambiguating polysemous words and recognizing semantic meanings such as expressions of fear, conversation style, etc. prevalent in concern-expressing tweets. Using embeddings with pre-encoded semantic meanings as features helps in performing few-shot learning i.e. training a good quality classifier with a small set of labeled data. The shared semantic meaning in embeddings ensures that the model learns to distinguish the semantic style and tone in tweets expressing concerns from others even when topical content varies. We used pre-trained Robust Bidirectional Encoder Representations from Transformers (RoBERTa) embeddings^25^ to transform words in tweets into numeric features. Twenty percent of the 3876 tweets were randomly sampled to create a test set, and the rest were used for training a logistic regression model. The trained model provided an AuC of 0.958 and an F1 score of 0.88 on the test data. We applied the trained model to 653 K tweets (from Sect "Data") to identify tweets expressing concerns.

Upon identifying tweets expressing concern, we performed regression analysis on tweets’ topic distributions (from Sect "Characterizing HPV discourse") to find topics correlated with “concern”. All topics with odds ratios > 1 and with confidence intervals of 95% were considered for further analysis. In parallel, we extracted 50 LDA topics from the training dataset and performed regression to identify topics correlated with concerns. The number of topics was set to 50 after analyzing topics’ quality.

### Leveraging GPT-4 for contextual topic labeling

A topic is a set of related words and requires additional analysis to derive an understandable and relevant theme. e.g. “*doctors, jab, decision, am, injured, daughter*”—> *adverse side-effects of vaccine/ vaccine injury.* Labeling topics can also help in connecting overlapping concerns (e.g. mistrust against pharmaceutical companies and adverse side effects). The labeling task demands an expert aware of the terminologies in HPV-related discourse and their implicit connotations.

A growing amount of literature supports the utility of language models as expert annotators with performance at par with humans^26^. We used a state-of-the-art language model, GPT-4 chat, and performed prompt engineering (See Table 1 for prompt and model parameters) to summarize the cluster of words in topics.

**Table 1:** Prompts used to label “Topic” and “Themes” using GPT 4 Chat.

Task	Prompt with an output example
Topic labeling	The following are topics associated with HPV vaccination on Twitter. The top words for each topic with the topic number are listed. For each topic, please find the relationship between words and conclude a topic with one short phrase. The output should only contain the topic number and its corresponding phrase < list of topics > Example: < effects, side, vax, adverse, look, reactions, safety, pharma, believe, studies, injuries, death, issues, reaction, serious > 1—Concerns and debates about vaccine side effects and safety
Theme labeling	The following are topics associated with HPV vaccination on Twitter. The topic number and top words for each topic are listed. Cluster all the topics into categories. Also, label each category based on the topics included in it < list of topics to be clustered > Example output: Category 1: HPV Vaccine Safety and Side Effects - 43: Lawsuit, merck, filed, #gardasil, over, time, recently, dangerous, challenging - 54: Effects, side, vax, adverse, reactions, safety, pharma, studies, injuries, death, issues, serious - 84: Shot, got, shots, flu, arm, second, rounds, last, booster, hurt, days, feel, bad, sore - 35: It's, think, would, say, isn't, doesn't, point, thing, actually, true, though, understand, can't, makes Note: Topics keywords are merged to identify overarching themes

The parameters are $temperature=0$, $to{p}_{p}=1$, $frequenc{y}_{penalty}=0$, and $presenc{e}_{penality}=0$.

## Results

### Performance evaluation

Almost half of the 653 K tweets were predicted as expressing a concern. Three human experts hand-coded randomly sampled 3 K tweets from the newly collected tweets dataset to evaluate the quality of the predictions. On a subset of 106 tweets, the kappa statistic for inter-rater agreement was 0.64 for (rater-1, rater-2), 0.6 for (rater-1, rater-3), and 0.6 for (rater 2, rater 3). Every tweet was handcoded by two coders and discrepancies in labels were resolved by consensus. 12.7% of 3 K tweets were hand-coded as expressing *concern*.

We obtained a recall of 71.4% and a precision of 31.2%. The objective of our study is to identify the new and evolving concerns against HPV vaccination, and we optimized our model for high recall which led to relatively poor precision. The model accurately captured the tone of worry in tweets; however, it also mislabeled the tweets expressing concern against the HPV disease as a concern. 28.6% of concern-expressing tweets were incorrectly predicted as not concern whereas 22.9% of non-concern tweets were incorrectly predicted as concern. The samples of misclassified tweets that were incorrectly predicted as a “concern” (i.e. False Positive) and incorrectly predicted as “not concern” (i.e. False negative) are provided in Table 2.

**Table 2:** Misclassified tweets: false positives and false negatives.

False positive
(1) Apparently, Big Morbidity and Mortality has its own spokespeople and advocates. And yes, HPV is VERY much < EMOJI > (2) < USER > You’ll punish teachers & children to make points with the Anti-Covid vaccine crowd? Why not stop vaccine mandates that protect children from chickenpox, Diptheria, flu, hepatitis, HPV, measles, mumps, polio, rubella, tetanus, and whooping cough? Talk about making points
False negative
(1) So correct. In Texas, when Rick Perry foolishly mandated Gardasil for kids, thankfully overridden by 100% (yes, 100 < EMOJI > of legislators. Buzz in Austin capital was it was for "HPV-Help Pay for Vioxx", Merck's prior disaster. < URL > (2) Significant fall in the number of students receiving the HPV vaccine concerning as only 53.6% of students received a first dose of the HPV vaccine in 2020 compared to over 80% in 2019 Read more on < URL > < URL >

### Topics associated with concern in HPV discourse

Forty-six topics were significantly correlated with concern expressing HPV discourse in the newly collected tweets (See Table 3). Lawsuits against Gardasil (“*lawsuit, merck, filed, behalf, #gardasil*”, OR = 3.36), personal experiences (“*women, she, her, pay, geico*”, OR = 2.05; “*where, especially, doctors, jab, decision*”, OR = 2.02), adverse side effects (“*effects, side, vax, adverse, look*”, OR = 1.73; “down, someone, off, bad, hope”, OR = 1.27) and vaccine mandates for adolescents (“*becuase, does would, those, remember*”, OR = 1.37; “*kids, them, vaccinated, covid, children*”, OR = 1.35) are the dominant themes behind vaccine hesitancy. Topics related to sexual health and STIs (“*hiv, herpes, too, aids, having*”, OR = 1.14), the effectiveness of HPV vaccination along with innate immunity to overcome HPV infection (“*immune, system, symptoms, body, its*”, OR = 1.09) are also debated. Additionally, topics (“*free, insurance, poor, cells, areas*”, OR = 1.05) discuss the availability of affordable vaccines for the poor and uninsured.

**Table 3:** Top 10 topics significantly correlated (p < 0.05) with concern in the new tweets dataset.

Theme	Topic label	Topic keywords	Odds ratio	CI (95%)
Adverse effects and controversies	Lawsuit filed against merck over gardasil	Lawsuit, merck, filed, behalf, #gardasil, its, over, time, recently, #merck, 13th, 25-year-old, dangerous, challenging, 19	3.357	[, ]
Personal experiences and opinions	Geico insurance case involving woman and car	Woman, she, her, pay, geico, car, 5.2, got, must, his, million, insured, caught, missouri, insurance	2.051	[2.049, 2.053]
	Doctors' decision on HPV vaccination and injured daughter	Where, especially, doctors, jab, decision, am, injured, daughter, speaking, section, catholic, teenage, unconscionable, hospitals, overturn	2.016	[2.013, 2.018]
Adverse effects and controversies	Adverse reactions and safety issues of HPV vaccine	Effects, side, vax, adverse, look, reactions, safety, pharma, believe, studies, injuries, death, issues, reaction, serious	1.729	[1.726, 1.732]
–	*Hospitals overwhelmed and kids' vaccination*	Take, never, hospitals, kids, made, overwhelmed, action, waiting, measles, heard, short, reported, #keungto, film, dipshit	1.662	[1.659, 1.665]
Personal experiences and opinions	Personal experience with daughter's HPV vaccination	She, her, daughter, me, ago, right, told, vax, thing, down, really, old, turned, mom, friend	1.403	[1.400, 1.406]
	Mandated HPV vaccinations in Texas schools	Because, does, would, those, remember, parents, mean, texas, perry, daughters, vaccinations, schools, saying, three, mandated	1.372	[1.369, 1.375]
	Parents' choice to vaccinate kids against COVID and HPV	kids, them, vaccinated, covid, children, want, child, parents, vax, give, vaccinate, same, choice, chance, catch	1.351	[1.348, 1.354]
	Government's decision to vaccinate boys and girls against HPV	Girls, boys, did, arm, punch, vaccinated, aged, both, vaccinating, 12, government, vaccinate, 14, catch-up, 13	1.317	[1.314, 1.320]
	Consent and vaccination of 12-year-old son	He, his, year, 12, old, him, son, given, said, consent, tried, form, proud, ground, UK	1.314	[1.311, 1.317]

Effect size is shown using odds ratios (ORs) along with 95% confidence intervals. Text in italics indicate GPT-4 chat generated label that was marked as incorrect by either of human annotators. Themes with “–” were not clustered under any themes. See Table S2 for the complete set of topics.

### Evaluating quality of topic labeling using GPT-4

Two human experts evaluated the quality of labels assigned by GPT 4 chat. The experts were asked to rate the correctness of the label on a 3-level Likert scale i.e. appropriate, somewhat appropriate, and not appropriate (See Table S3 for annotation guidelines). Out of 46 topics (OR > 1 and p < 0.05), only ten (21.7%) topic labels generated using prompt-1 were marked as “not appropriate” by either of the annotators, whereas all clustered theme labels generated using prompt-2 were marked as “appropriate” (i.e., all topics fall under this theme) or “somewhat appropriate” (i.e., not all topics fall under this theme but the majority do). These labels were also generated for the training tweets dataset^8^; three (17.6%) out of 17 topic labels (OR > 1 and p < 0.05) generated using prompt-1 were marked as “not appropriate” by either of the annotators. In contrast, all theme labels were marked as “appropriate” or “somewhat appropriate.” It is worth noting that the GPT-4 chat tends to over-generalize when clustering topics and labeling themes, e.g. topics related to vaccine mandates were assigned the theme “Personal Experiences and Opinions,” which is not completely incorrect but lacks the precise concern.

### Pre and post-2020: evolution of concerns against HPV vaccination

The themes in tweets pre-2020 are (a) Adverse Effects and Controversies (“*#vaccineinjury, after, #study*)*:, case, et*”, OR = 1.45; “*after, gardasil, dr, expert, harper*”, OR = 1.43), (b) Vaccine Efficacy and Disease Prevention (“*may, lesions, disease, it's, merck's*”, OR = 1.21), (c) Vaccine Mandates and Parental Concerns (“*mandate, school, parents, #hpvvax, vaccines*”, OR = 1.18), and (d) Personal Experiences and Opinions (“*they, his, was, my, he, there*”, OR = 1.14) (See Table 4).

**Table 4:** Top 10 topics significantly correlated (p < 0.05) with concern in the training dataset i.e., tweets posted pre 2020^8^.

Theme	Topic label	Topic keywords	Odds ratio	CI (95%)
Adverse effects and controversies	Case studies of vaccine injury post HPV vaccination	#vaccineinjury, after, #study):, case, et, #adem, days, al, #wakeup	1.452	[1.413, 1.492]
	Side effects and deaths post Gardasil vaccination	After, gardasil, dr, expert, harper, diane, no, #vaxxed, side, dies	1.428	[1.389, 1.468]
	Adverse events in hpv vaccine clinical study	Trials, #study, adverse, review, serious, events, after, did, randomized, its	1.399	[1.360, 1.438]
	Vaccine injury in 13-year-olds post HPV vaccination	After, year, #vaccineinjury, old, 13, into, kids, shot, part, being	1.377	[1.338, 1.416]
	*Side effects in teens after HPV vaccination*	After, effects, teens, side, over, #vaccineswork, receiving, #flu, court, #measles	1.240	[1.203, 1.277]
Vaccine efficacy and disease prevention	Potential increase in lesions and disease post Merck's HPV vaccination	May, lesions, disease, it's, merck's, up, without, increases, own, 44.6	1.213	[1.176, 1.250]
Vaccine mandates and parental concerns	School mandates and parental concerns over HPV vaccination	Mandate, school, parents, #hpvvax, vaccines, bill, want, #nonymandates, mandatory, trying	1.184	[1.148, 1.221]
Vaccine efficacy and disease prevention	Link between ovarian failure and HPV vaccination	Call, make, today, failure, following, ovarian, link, premature, between, event	1.154	[1.118, 1.190]
	Concerns over deaths and injuries caused by HPV vaccination	Cause, deaths, need, injured, already, start, going, #ny, think, #senatorhoylman	1.148	[1.113, 1.184]
Personal experiences and opinions	Personal experiences and stories related to HPV vaccination	They, his, was, my, he, there, who, had, were, them	1.139	[1.104, 1.175]

Effect size is shown using odds ratios (ORs) along with 95% confidence intervals. Text in italics is the GPT-4 chat generated label that was marked as incorrect by either of the human annotators. See Table S1 for the complete set of topics.

In tweets post 2020, topics “adverse effects and controversies” (“*lawsuit, merck, filed, behalf, #gardasil*”, OR = 3.36) and “Vaccine Mandates and Parental Concerns” (“*because, does, would, those, remember*”, OR = 1.32) remained the top concerns. Personal experiences centered on parent’s consent and vaccine mandates are discussed profusely. We also observe unseen themes i.e. HPV and Women's Health (“*girls, india, gates, bill, africa, foundation*”, OR = 1.3), HPV Vaccine Development and Market (“*big, money, way, keep, where, daughters*”, OR = 1.22; “*market, data, pdsb, china, top*”, OR = 1.02), and Sexual Health and STIs (“*hiv, herpes, too, aids, having*”, OR = 1.14; “*hiv, herpes, list, syphilis, gonorrhea*”, OR = 1.09) and body immunity (“*immune, system, symptoms, body, its*”, OR = 1.09) (See Table S2).

Overall, the discussion on HPV vaccination has become more personal in the past few years, with more individuals questioning the HPV vaccine mandates for school children. Consequently, we also see more tweets sharing personal experiences and increasing parents’ reluctance towards the HPV vaccine for their children.

## Discussion

Disproving misinformation on social media is a challenge, often intensified by the lack of explicit information countering the false claims, thereby strengthening individuals' beliefs. Particularly concerning HPV vaccine misinformation, posts of this nature tend to receive higher "likes" compared to pro-vaccine content^20^, amplifying their visibility among wider audiences. Amid the COVID-19 pandemic, vaccination-related concerns surged due to rapidly changing guidelines. Healthcare professionals and policymakers encounter a significant challenge in compiling and comprehending the diverse array of vaccine-related concerns causing hesitancy. People's tendency to seek information online regarding potential adverse effects, and often finding search results affirming their fears, has further complicated the vaccination landscape.

The unfiltered access to first-hand public opinions on social media presents an opportunity to learn and address concerns that might otherwise go unnoticed. In the past, topical analyses coupled with human supervision were used for collating reasons behind vaccine hesitancy^27,28^. The proposed pipeline revealed vaccine safety, vaccine effectiveness, and mistrust due to vaccine mandates as major concerns in pre 2020 tweets; this is aligned with prior findings examining social media posts from a similar time frame^16^. Increasing negative anecdotal reports influence parents’ decision to vaccinate their children^7^. The reasons behind vaccine hesitancy also evolve with the socio-political environment (e.g. increased mistrust against government and healthcare institutions during COVID-19) and health policies (e.g. mandating vaccines). Our approach can be easily adapted to detect multilingual concerns at the desired region level such as country, state, county, etc. with minimal human supervision.

There are several limitations to our approach that need to be addressed. First, our study was limited to tweets in English and did not cover concerns of non-English speakers. Second, we limited our analysis to textual posts however, multimodal content such as memes on X and other social media platforms such as Tiktok and YouTube are also of interest^29^. Third, not all expressions of vaccine hesitancy manifest in overtly negative or explicit concerns. Our model struggles to detect obscure concerns against vaccination^30,31^. Below are a few examples that were misclassified:

*“RT* <*USER*>*: I thought* <*EMOJI*> *aged out of the HPV vaccines but you can them until* <*EMOJI*> *45! Expensive, though.”*

*Here,* the writer does not have a negative stance against the HPV vaccine, however, the cost of the vaccine is the concern.

“<*USER*> *HPV isn't even that dangerous, and it doesn't show up in males. Even if that WERE true, there is absolutely no way Vinny could've known he had it because there's no male HPV test, from what I understand.*”

Here, the writer has projected HPV as a low-risk illness and does not have an explicit negative stance against vaccination or tests.

Tweets can also have a sarcastic tone; a negative message is conveyed with a positive undertone.

*“Thanks* <*USER*> *- your dangerous and ineffective HPV vaccine is doing a great job!* <*EMOJI*> <*URL*>*”*

*“RT* <*USER*>*: How did that HPV vaccine go again? How many young girls were paralyzed by this perfectly safe and vitally necessary vaccine?* <*EMOJI*>*”*

Or, tweets plainly stating facts or news that have a negative undertone.

*“RT* <*USER*>*: More than 25% of parents in 2019 refused the HPV vaccine for their child, up from 5% in 2008, showing "disinformation* <*EMOJI*>*”*

To conclude, automating the detection and monitoring of vaccination-related concerns is a complex task that could immensely benefit from advancing natural language processing. LLMs could help examine obscure motivations^32^ behind vaccine hesitancy. More research is needed to expand digital social listening to multimodal content.

### Supplementary Information

Supplementary Tables.

## Data Availability

The datasets generated and/or analyzed during the current study are available from the corresponding author upon reasonable request.
