# Supplementary material for: Detecting and monitoring concerns against HPV vaccination on social media using large language models
Source: Sci Rep. 2024 Jun 21;14:14362. doi: 10.1038/s41598-024-64703-3 (PMC11192875; doi:10.1038/s41598-024-64703-3)
Supplement: Supplementary file 1 — Supplementary Tables. [file 41598_2024_64703_MOESM1_ESM.pdf]

Table S1. Topics significantly correlated (p<0.05) with concern in the training dataset (Kornides et al. 2022). Effect size is shown using odds ratios (ORs) along with 95% CIs. Text in red is the GPT-4 generated label that was marked as incorrect by either of the human annotators.

Theme	Topic Label	Topic Keywords	odds ratio	CI (95%)
Adverse Effects and Controversies	Case Studies of Vaccine Injury Post HPV Vaccination	#vaccineinjury, after, #study,):, case, et, #adem, days, al, #wakeup	1.452	[1.413, 1.492]
	Side Effects and Deaths Post Gardasil Vaccination	after, gardasil, dr, expert, harper, diane, no, #vaxxed, side, dies	1.428	[1.389, 1.468]
	Adverse events in hpv vaccine clinical study	trials, #study, adverse, review, serious, events, after, did, randomized, its	1.399	[1.360, 1.438]
	Vaccine Injury in 13-Year-Olds Post HPV Vaccination	after, year, #vaccineinjury, old, 13, into, kids, shot, part, being	1.377	[1.338, 1.416]
	Side Effects in Teens After HPV Vaccination	after, effects, teens, side, over, #vaccineswork, receiving, #flu, court, #measles	1.240	[1.203, 1.277]
Vaccine Efficacy and Disease Prevention	Potential Increase in Lesions and Disease Post Merck's HPV Vaccination	may, lesions, disease, it's, merck's, up, without, increases, own, 44.6	1.213	[1.176, 1.250]
Vaccine Mandates and Parental Concerns	School Mandates and Parental Concerns Over HPV Vaccination	mandate, school, parents, #hpvvax, vaccines, bill, want, #nonymandates, mandatory, trying	1.184	[1.148, 1.221]
Vaccine Efficacy and Disease Prevention	Link Between Ovarian Failure and HPV Vaccination	call, make, today, failure, following, ovarian, link, premature, between, event	1.154	[1.118, 1.190]
	Concerns Over Deaths and Injuries Caused by HPV Vaccination	cause, deaths, need, injured, already, start, going, #ny, think, #senatorhoylman	1.148	[1.113, 1.184]
Personal Experiences and Opinions	Personal Experiences and Stories Related to HPV Vaccination	they, his, was, my, he, there, who, had, were, them	1.139	[1.104, 1.175]
Adverse Effects and Controversies	Controversy Over Increase in Adverse Effects Reports Post HPV Vaccination	increase, rates, rise, age, reports, study, groups, adverse, effects, controversy	1.133	[1.099, 1.169]
Personal Experiences and Opinions	Questions and Doubts About Gardasil HPV Vaccine	up, making, too, where, gardasil, few, yet, merck, never, which	1.122	[1.087, 1.157]
	Importance of HPV Vaccinations and Link to Diagnosis	dr, also, vaccinations, which, was, linked, diagnosis, young, here, importance	1.101	[1.067, 1.136]
Vaccine Efficacy and Disease Prevention	Testing of HPV Vaccines on Children and Comparison to Other Vaccines	testing, children, #hepatitis, group, control, was, did, #hiv, use, like	1.099	[1.065, 1.134]
	Evidence of Autoimmune Potential in First Approved HPV Vaccine	first, has, according, approved, potential, autoimmune, #informedconsent, here, evidence, its	1.097	[1.063, 1.132]
Personal Experiences and Opinions	Questions and Discussions About HPV Vaccination	who, why, what, should, right, only, see, talk, doctor, vax	1.060	[1.027, 1.094]
Vaccine Mandates and Parental Concerns	Parental Opinions on Vaccinating Their Children Against HPV	vaccines, they, should, parents, their, children, them, one, don't, need	1.053	[1.020, 1.086]

Table S2. Topics significantly correlated (p<0.05) with concern in the new tweets dataset. Effect size is shown using odds ratios (ORs) along with 95% CIs. Text in red is the GPT-4 generated label that was marked as incorrect by either of human annotators. Themes with "-" were not clustered under any themes.

Theme	Topic Label	Topic Keywords	odds ratio	CI (95%)
Adverse Effects and Controversies	Lawsuit Filed Against Merck Over Gardasil	lawsuit, merck, filed, behalf, #gardasil, its, over, time, recently, #merck, 13th, 25-year-old, dangerous, challenging, 19	3.357	[,]
Personal Experiences and Opinions	Geico Insurance Case Involving Woman and Car	woman, she, her, pay, geico, car, 5.2, got, must, his, million, insured, caught, missouri, insurance	2.051	[2.049, 2.053]
	Doctors' Decision on HPV Vaccination and Injured Daughter	where, especially, doctors, jab, decision, am, injured, daughter, speaking, section, catholic, teenage, unconscionable, hospitals, overturn	2.016	[2.013, 2.018]
Adverse Effects and Controversies	Adverse Reactions and Safety Issues of HPV Vaccine	effects, side, vax, adverse, look, reactions, safety, pharma, believe, studies, injuries, death, issues, reaction, serious	1.729	[1.726, 1.732]
-	Hospitals Overwhelmed and Kids' Vaccination	take, never, hospitals, kids, made, overwhelmed, action, waiting, measles, heard, short, reported, #keungto, film, dipshit	1.662	[1.659, 1.665]
Personal Experiences and Opinions	Personal Experience with Daughter's HPV Vaccination	she, her, daughter, me, ago, right, told, vax, thing, down, really, old, turned, mom, friend	1.403	[1.400, 1.406]
	Mandated HPV Vaccinations in Texas Schools	because, does, would, those, remember, parents, mean, texas, perry, daughters, vaccinations, schools, saying, three, mandated	1.372	[1.369, 1.375]
	Parents' Choice to Vaccinate Kids Against COVID and HPV	kids, them, vaccinated, covid, children, want, child, parents, vax, give, vaccinate, same, choice, chance, catch	1.351	[1.348, 1.354]
	Government's Decision to Vaccinate Boys and Girls Against HPV	girls, boys, did, arm, punch, vaccinated, aged, both, vaccinating, 12, government, vaccinate, 14, catch-up, 13	1.317	[1.314, 1.320]
	Consent and Vaccination of 12-Year-Old Son	he, his, year, 12, old, him, son, given, said, consent, tried, form, proud, ground, uk	1.314	[1.311, 1.317]
	Efforts to Stop the Spread of HPV	did, which, me, getting, going, spread, stop, disease, everyone, please, strains, possibly, help, refuse, accept	1.310	[1.307, 1.313]
	Parents' Consent for School Vaccination	parents, school, vaccinated, consent, these, without, she, her, today, days, young, feel, niece, diagnosed, express	1.308	[1.305, 1.311]
	Refusal of HPV Vaccination in School	refused, where, jab, class, asked, him, son, teacher, pulled, free, good, returned, love, working, find	1.305	[1.302, 1.308]
HPV and Women's Health	Bill Gates Foundation's HPV Vaccine Trials in India and Africa	girls, india, gates, bill, africa, foundation, trials, his, used, much, injection, knowledge, poor, tribal, didnt	1.292	[1.289, 1.295]
Personal Experiences and Opinions	Negative Reactions to HPV Vaccine Commercial	down, someone, off, bad, hope, oh, fuck, maybe, fucking, looking, lol, coming, ass, commercial, yeah	1.270	[1.267, 1.273]
	Personal Thoughts and Experiences with HPV Vaccine	i'm, too, think, am, really, didn't, i've, back, ok, going, sure, though, off, came, thought	1.263	[1.260, 1.266]
	Experience with HPV and Flu Shots	shot, got, shots, flu, arm, second, then, rounds, last, booster, hurt, days, feel, bad, sore	1.256	[1.253, 1.259]
	Personal Opinions and Doctor-Patient Communication about HPV Vaccine	me, even, want, say, person, anything, tell, others, doctor, fact, ask, wanna, partner, matter, older	1.228	[1.225, 1.231]
-	Big Pharma and HPV Vaccine Delivery in Lagos	big, money, way, keep, where, daughters, herbs, plexus, come, lagos, choroid, pharma, paid, sending, delivery	1.217	[1.214, 1.220]
Vaccination Policies and Mandates	Mandatory HPV Vaccination for School Attendance	school, us, needed, children, bill, mandate, attend, state, mandates, college, mandatory, outreach, regarding, ny, under	1.194	[1.191, 1.197]
-	Comparison of HPV Vaccine with Other Vaccines	covid, flu, vax, pox, shingles, only, chicken, i'm, wait, oh, lifetime, jab, went, here's, same	1.181	[1.178, 1.183]
-	Dr. Epstein's Thoughts on HPV Cure	cure, me, dr, always, never, thought, he, growing, saw, teenagers, reality, great, email, epstein, com	1.179	[1.176, 1.182]
-	Role of HPV Vaccine in Preventing Diseases	prevents, right, since, literally, gonna, should, me, focused, box, found, clinic, soap, folks, cure, role	1.177	[1.174, 1.179]
-	Promotion of HPV Vaccination for Children	today, says, children, any, stop, dr, promoting, kind, because, first, getting, department, got, age, under	1.166	[1.163, 1.168]

-	Diphtheria, Tetanus, and Other Diseases Prevented by Vaccination	4x, diphtheria, times, several, tetanus, regimen, rip-off, diseases, taking, influenza, down, vaccinations, mmr, such, pertussis	1.155	[1.153, 1.158]
-	Death Caused by HPV	only, caused, woman, died, sister, ago, december, 16th, pepper, hawked, first, love, second, frame, among	1.151	[1.148, 1.154]
-	Note on Spreading Germs in College	000, please, shit, 1,000, germs, salt, gram, pinch, before, note, lick, asskrim, college, spread, that's	1.145	[1.142, 1.147]
Personal Experiences and Opinions	Personal Opinions on HPV Vaccine	it's, think, would, that's, say, isn't, doesn't, point, thing, actually, true, though, understand, can't, makes	1.141	[1.139, 1.144]
Sexual Health and STIs	High Rate of HIV, Herpes, and Other STDs	hiv, herpes, too, aids, having, way, chlamydia, unprotected, high, rate, side, pieces, hoes, damn, gonorrhea	1.139	[1.137, 1.142]
-	Lawsuit Over Unauthorized Use of Black Woman's Cells for Research	family, were, research, taken, without, papilloma, black, cells, woman, whose, genital, consent, enough, firm, suing	1.111	[1.108, 1.113]
Personal Experiences and Opinions	Australian Mandates on HPV Vaccine	see, series, australian, together, place, again, easy, mandates, round, death, put, unisa, nobody, hear, proof	1.105	[1.103, 1.108]
Sexual Health and STIs	List of STDs Including HIV, Herpes, and Gonorrhea	hiv, herpes, list, syphilis, gonorrhea, chlamydia, these, tested, diseases, sti, different, hsv, std's, exhaustive, malaria	1.093	[1.090, 1.095]
HPV and Women's Health	Immune System's Response to HPV	immune, system, symptoms, body, its, does, own, away, clear, develop, usually, someone, however, strains, already	1.089	[1.086, 1.092]
HPV and Women's Health	Data on HPV Vaccine's Effectiveness in Preventing Deaths	only, immunity, prevents, data, let, preventable, forms, thus, whether, deaths, says, clearly, daughters, studies, refuse	1.063	[1.060, 1.066]
HPV and Women's Health	COVID and HPV Infections Prevented by Vaccines	which, forget, prevents, covid, amazing, due, pretty, gotten, infections, #vaccine, 1st, #gardasil, day, included, saw	1.059	[1.057, 1.062]
HPV and Women's Health	Free HPV Vaccinations for the Poor and Uninsured	free, insurance, poor, cells, areas, care, birthday, injections, part, offer, henrietta, medical, lacks, celebrate, providing	1.055	[1.053, 1.058]
HPV and Women's Health	Rise of HPV Infections Amid Pandemic	pandemic, there's, amid, could, work, zur, hausen, papilloma, harald, give, rise, despite, led, infections, his	1.054	[1.051, 1.056]
-	Antivax Movement and HPV Vaccine	wrong, were, work, remember, hey, antivax, something, movement, swore, receiving, she, knew, weeks, give, eventually	1.040	[1.038, 1.043]
Personal Experiences and Opinions	Laura Brennan's Advocacy for HPV Vaccine	today, her, laura, would, brennan, last, life, birthday, 28th, months, dedicated, advocate, 1/4, working, family	1.039	[1.037, 1.042]
Vaccination Policies and Mandates	Importance of HPV Vaccination for School Students	school, year, students, vaccinations, missed, important, child, remember, affect, grade, covid19, groups, clinics, instead, schools	1.038	[1.036, 1.041]
Personal Experiences and Opinions	Personal Experience with Side Effects of HPV Vaccine	work, back, last, hard, try, wait, pain, body, love, huge, effect, eye, look, crazy, can't	1.029	[1.027, 1.032]
Vaccination Policies and Mandates	National Prevention Program for HPV and COVID-19	covid-19, effects, side, national, prevention, world, program, immunization, routine, increase, working, community, japan, map, support	1.025	[1.022, 1.027]
Vaccination Policies and Mandates	Use of MMR, Flu, and Other Vaccines	mmr, doses, flu, shots, months, hep, dtap, hib, use, dose, hepb, booster, jabs, pcv, etc	1.025	[1.022, 1.027]
HPV Vaccine Development and Market	Market Data on HPV Vaccine Trials in China	market, data, pdsb, china, top, trial, company, phase, treat, big, developed, face, combo, download, billion	1.020	[1.018, 1.023]
Sexual Health and STIs	Sexual Health and HPV Testing	sexual, any, you're, tests, partner, use, does, simple, god, man, condoms, anyone, gender, community, support	1.009	[1.007, 1.012]
Personal Experiences and Opinions	Personal Experience and Support During HPV Vaccination	need, different, her, talk, until, play, yet, happy, always, loved, during, went, visit, provide, experience	1.006	[1.003, 1.008]

Table S3 Annotation guidelines	
**Task**	**Instruction to Annotators.**
Topic Labeling	Read the Word Cluster (topic) in Column A and the machine-generated label in Column C, the task is to determine if the machine-generated label is appropriate or not. You are encouraged to refer to an example tweet (in Column B) to gather more context on the topic.
Theme Labeling	Task: Read the text (label) in the highlighted cell. Refer to the topics (word cluster) under the label and determine if the given label is appropriate for the topics under the label. If a topic is inappropriate, enter -1 in column B.

Table S4 Automatic performance evaluation for topic modeling

Coherence metric	Number of Topics		
	50	100	150
U_Mass	-5.649	-6.177	-6.587
C_V	0.493	0.506	0.509
C_UCI	-1.317	-1.489	-1.786
C_NPMI	0.011	0.013	0.011
